# EXPERIENCES AND BARRIERS TO SUCCESS FOR MID- AND LATER-LIFE COLLEGE STUDENTS: APPLYING A GERONTOLOGICAL LENS

**DOI:** 10.1093/geroni/igz038.2468

**Published:** 2019-11-08

**Authors:** Phyllis Cummins, Annabelle Arbogast, Kathryn McGrew, Peter Bahr

**Affiliations:** 1 Scripps Gerontology Center, Miami University, Oxford, Ohio, United States; 2 Miami University, Oxford, Ohio, United States; 3 Miami University, Scripps Gerontology Center, Oxford, Ohio, United States; 4 University of Michigan, Michigan, United States

## Abstract

Adult students have emerged as a key population of interest within higher education as states and institutions strategize to meet postsecondary attainment goals. However, much of the previous research on non-traditional age college students has collapsed all students age 25 and older into a single category, glossing over important age and life stage differences. Using a gerontological lens, this paper examines experiences and barriers encountered by mid-and later-life (MLL) students (age 40 and older) attending community colleges. We report qualitative findings from a mixed-methods study of MLL students in Ohio community colleges, funded by the Institute of Education Sciences. Based on thematic analysis of interviews and focus groups with students, faculty, staff, and administrators at 23 colleges, we identify multiple dimensions of age and aging that each play a meaningful role in shaping MLL students’ community college experiences and outcomes. Additionally, we provide an in-depth profile of MLL students—including their educational and work trajectories, reasons for enrolling, and experiences in community colleges—that can help colleges better recruit and serve this segment of the adult student population. MLL students face both unique and common barriers that colleges can address at the classroom, program, and institution levels. Implications for research, policy, and practice are discussed.

